# Synthetic incorporation of Nile Blue into DNA using 2′-deoxyriboside substitutes: Representative comparison of (*R*)- and (*S*)-aminopropanediol as an acyclic linker

**DOI:** 10.3762/bjoc.6.13

**Published:** 2010-02-09

**Authors:** Daniel Lachmann, Sina Berndl, Otto S Wolfbeis, Hans-Achim Wagenknecht

**Affiliations:** 1 University of Regensburg, Institute for Organic Chemistry, 93040 Regensburg, Germany; 2 University of Regensburg, Institute for Analytical Chemistry, Chemo- and Biosensors, 93040 Regensburg, Germany

**Keywords:** cycloaddition, fluorescence, glycol, oligonucleotide, phenoxazinium, phosphoramidite

## Abstract

The Nile Blue chromophore was incorporated into oligonucleotides using “click” chemistry for the postsynthetic modification of oligonucleotides. These were synthesized using DNA building block **3** bearing an alkyne group and reacted with the azide **4**. (*R*)-3-amino-1,2-propanediol was applied as the linker between the phosphodiester bridges. Two sets of DNA duplexes were prepared. One set carried the chromophore in an A-T environment, the second set in a G-C environment. Both were characterized by optical spectroscopy. Sequence-dependent fluorescence quenching was applied as a sensitive tool to compare the stacking interactions with respect to the chirality of the acyclic linker attachment. The results were compared to recent results from duplexes that carried the Nile Blue label in a sequentially and structurally identical context, except for the opposite chirality of the linker ((*S*)-3-amino-1,2-propandiol). Only minor, negligible differences were observed. Melting temperatures, UV–vis absorption spectra together with fluorescence quenching data indicate that Nile Blue stacks perfectly between the adjacent base pairs regardless of whether it has been attached via an *S*- or *R*-configured linker. This result was supported by geometrically optimized DNA models.

## Introduction

Chemical bioanalysis and imaging of nucleic acids require synthetic incorporation of fluorescent DNA probes for an optical readout [1–9]. A large variety of organic fluorophores can be incorporated routinely at specific positions within the oligonucleotide using standard phosphoramidite DNA chemistry and commercially available DNA building blocks. However, problems can arise if labels or probes are chemically incompatible with the conditions of DNA synthesis, or if the stepwise synthesis of the corresponding DNA building block fails. So-called postsynthetic modifications can overcome these limitations and allow the synthetic modification of a presynthesized oligonucleotide carrying a special functional group [10]. Bioorthogonality is required for these ligation reactions in that both the functional group of the oligonucleotides and the functional group of the modifier should not be present in typical biomolecules and should react selectively with each other [11]. Over the last five years the Huisgen–Meldal–Sharpless “click” ligation strategy has become an important strategy for postsynthetic labeling of DNA [12–13]. Huisgen described first the [2+3]-cycloaddition between alkynes and azides yielding 1,2,3-triazoles [14]. The utility of this reaction as a bioligation method has grown incredibly after Meldal [15] and – almost at the same time – Sharpless [16] had reported that the addition of Cu(I) led to a significant increase in the reaction rate and in regioselectivity. This type of “click” chemistry matches the requirements of bioorthogonality since both two functional groups, alkyne and azide, are typically not present in biopolymers and react selectively with each other in aqueous solutions.

The “click” ligation can avoid the time-consuming synthesis of phosphoramidites as DNA building blocks which is especially important for brightly emitting fluorophores that are not compatible with the acidic, oxidative or basic conditions of automated DNA phosphoramidite chemistry and/or DNA workup. We recently presented the postsynthetic incorporation of Nile Blue and a coumarin dye as representatives of base-labile fluorophores by the “click” ligation strategy [17]. Several other fluorophores (spanning the whole visible spectrum) for use in “click” conjugation have been reported meanwhile [18]. The dye carrying the azide group was reacted with an alkyne group that was incorporated into the oligonucleotides as a nucleotide substituent. (*S*)-3-Amino-1,2-propanediol was used as an acyclic linker and substitute for the 2′-deoxyriboside between the phosphodiester bridges. Similar propanediol derivatives have been used extensively as alternative and simplified phosphodiester linkers in the 1990s [19–23], and have been further explored for glycol nucleic acid (GNA) [24–26] twisted intercalating nucleic acids (TINA) [27–28], and by our group for fluorescent DNA base substitutions by ethidium [29–30], indole [31–32], thiazole orange [33–34], perylene bisimide [35–36] and phenothiazine [37]. This 2′-deoxyriboside substitution provides high chemical stability and conformational flexibility for the chromophore to intercalate.

**Scheme 1:**
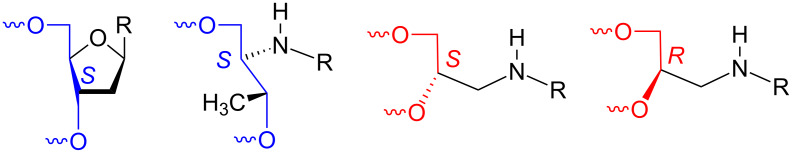
Chirality of C-3 of natural 2′-deoxyribofuranosides (left) in comparison with the acyclic D-threoninol [38] and (*S*)-[17] and (*R*)-3-amino-1,2-propanediol linker of this study (to the right).

The major difference between the 3-amino-1,2-propanediol linker and the 2′-deoxyribofuranoside is the number of carbon atoms between the phosphodiester bridges in the corresponding modified oligonucleotides which has been reduced from 3 (in normal nucleosides) to 2 (Scheme 1). However, we have shown that the ethidium dye connected to D-threoninol as a linker bearing three carbon atoms between the phosphodiester bridges has nearly the same optical properties as the shorter (S)-3-amino-1,2-propanediol linker [38]. It is important to point out, however, that chirality of the acyclic linker system has proven to be an important aspect in terms of chromophore stacking orientation [39–40], oligonucleotide function [41] and duplex formation [42]. Komiyama et al. were able to show that D- and L-threoninol as alternative acyclic linker systems act differently as 2′-deoxyribose substitutes by influencing the stacking orientation of an attached azobenzene dye. The configuration of the linker decides if the dye protrudes towards the major or minor groove, which subsequently leads to an enhanced or diminished stability of the whole DNA duplex [42]. Herein, we want to explore how important the chirality of the 3-amino-1,2-propanediol linker is with respect to the optical properties of an attached fluorophore. We chose Nile Blue as the fluorescent probe for these experiments since the redox properties of this phenoxazinium label exhibit a potential sufficient for photooxidizing guanines in the sequential neighborhood [17]. As a result, fluorescence quenching is observed in DNA and it is important to note that we decided to use this property as a sensitive tool to compare the electronic interactions of the phenoxazinium chromophore with the DNA base stack in order to evaluate the role of the chirality of the 3-amino-1,2-propanediol linker.

## Results and Discussion

**Scheme 2:**
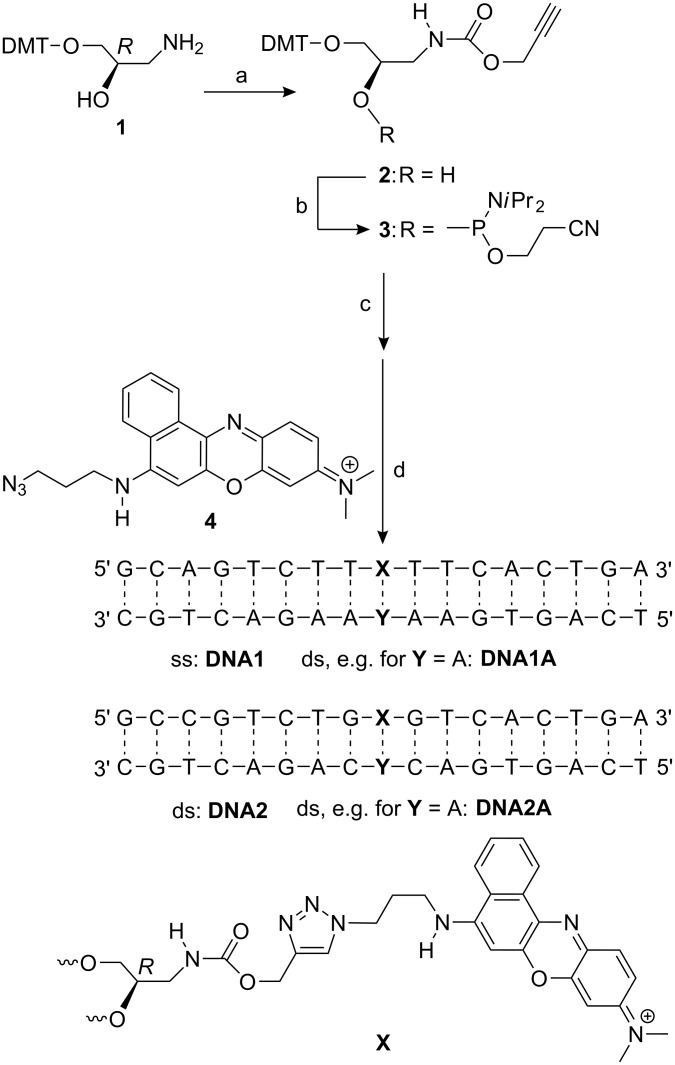
Synthesis of the *R*-configured DNA building block **3** and postsynthetic click ligation of the Nile Blue-modified single strands (ss) **DNA1** and **DNA2** that form the double strands (ds) **DNA1Y** and **DNA2Y** with the counterparts **Y** = A, T, C, G; a) 2-propyn-1-ol, 1,1′-carbonyldiimidazole, DMF, r.t., 27 h; 44%; b) 2-cyanoethyl-*N*,*N*-diisopropylchlorophosphoramidite, EtN(*i*Pr)_2_, CH_2_Cl_2_, r.t., 3 h.

The DNA building block ethynyl (2*R*)-3-(4,4′-dimethoxytrityl)-2-[(2-cyanoethoxy)(diisopropylamino)phosphinooxy]propylcarbamate (**3**) contains the propargyl group attached to the glycol part by a carbamate function (Scheme 2). In comparison to our earlier synthetic protocols for incorporation of ethidium [29–30], indole [31] and phenothiazine[37] the NH group of the carbamate is less nucleophilic and need not be protected during phosphoramidite synthesis. This facilitates the preparation of DNA building blocks as we have shown with indole [32] and thiazole orange [33–34]. DMT-protected (*R*)-3-amino-1,2-propanediol **1** as a precursor was synthesized according to literature [29,43]. The hydroxy function of commercially available 2-propyn-1-ol was converted into an activated ester by 1,1′-carbonyldiimidazole and subsequent acylation gave conjugate **2** in 44% yield. The synthesis of phosphoramidite **3** was finished by standard procedures, and **3** was applied directly for automated DNA synthesis. Presynthesized oligonucleotides for **DNA1** and **DNA2** were reacted with the Nile Blue azide **4** according to our “click”-type ligation protocol, worked up and purified according to literature [17]. The two Nile Blue-modified oligonucleotides **DNA1** and **DNA2** were identified by ESI mass spectrometry, quantified by UV–vis absorption and characterized by fluorescence spectroscopy (including quantum yields) and measurement of melting temperatures (*T*_m_). DNA duplexes are formed by heating in the presence of 1.2 equiv of unmodified counterstrands at 90 °C for 10 min followed by slow cooling to r.t. The two representative oligonucleotides, **DNA1** and **DNA2**, expose the blue phenoxazinium chromophore to two different variations in the neighborhood: (i) the dye was placed either in an A-T environment (**DNA1Y**) or next to G-C base pairs (**DNA2Y**), and (ii) within each duplex set, **DNA1Y** and **DNA2Y**, the base opposite to the chromophore site was varied (**Y** = A, T, C or G).

Representatively, we compared melting temperatures of the Nile Blue-modified duplexes **DNA1A** and **DNA2A** with the corresponding unmodified DNA sequences that bear a standard T instead of the Nile Blue chromophore (Table 1). The replacement of the natural 2′-deoxyribofuranoside by the *R*-configured acyclic glycol linker results in a decrease of thermal stability by 3.4 °C in **DNA1A** and 1.3 °C in **DNA2A**. Destabilization of the corresponding duplexes with the *S*-configured linker was similar [16].That means that there is no significant difference (Δ*T*_m_ ≤ 2.5 °C) between the *S*- and *R*-configured acyclic linker that attaches Nile Blue to DNA. Obviously, both configurations induce a rather small destabilization, but do not exhibit any clear dependence of the thermal stability of the DNA duplex on chirality. In comparison with the literature our results are remarkable with respect to three different aspects. (i) Our results stand in contrast to experiments with D- or L-threoninol [42], the alternative acyclic linker mentioned above, where chirality decided about duplex stability. (ii) Single glycol modifications typically yield a strong destabilization [19–23]. For instance, incorporation of indole as a single base surrogate into oligonucleotides resulted in strong destabilization of the DNA duplexes (ca. −12 °C) [32]. Our results with Nile Blue indicate that the interactions of the hydrophobic chromophore with the adjacent base pairs regain some of the lost thermal stability that is caused by the glycol linker. (iii) The melting temperatures of Nile Blue-modified duplexes are similar within each duplex set, **DNA1Y** or **DNA2Y**. This is typical for chromophores as base surrogates [26–36] since they do not exhibit any preferential base pairing properties.

**Table 1:** Melting temperatures (*T*_m_) and quantum yields (Φ_F_) of duplex sets **DNA1Y** and **DNA2Y** bearing the *R*-configured linker in comparison to the corresponding values with the *S*-configured linker [17].

Duplex	*R* configuration		*S* configuration [17]		Differences *R*–*S*	
	*T*_m_ (°C)	Φ_F_	*T*_m_ (°C)	Φ_F_	Δ*T*_m_ (°C)	ΔΦ_F_

**DNA1** (ss)	–	0.15	–	0.16	–	−0.01
**DNA1A**	59.1 (−3.4)^a^	0.17	56.6 (−5.9)^a^	0.15	+2.5	+0.02
**DNA1T**	58.5	0.22	60.4	0.19	−1.9	+0.03
**DNA1C**	60.7	0.21	58.5	0.22	+2.2	−0.01
**DNA1G**	59.1	0.05	59.8	0.06	−0.7	−0.01
**DNA2** (ss)	–	0.04	–	0.05	–	−0.01
**DNA2A**	66.7 (−1.3)^b^	0.02	65.5 (−2.5)^b^	0.02	+1.2	<0.01
**DNA2T**	64.2	0.02	66.5	0.02	−2.3	<0.01
**DNA2C**	67	0.02	67.5	0.01	−0.5	<0.01
**DNA2G**	66.8	0.01	67.7	0.01	−0.9	<0.01

^a^In comparison to an unmodified duplex with *T* instead of the chromophore: *T*_m_ = 62.5 °C [17]. ^b^In comparison to an unmodified duplex with *T* instead of the chromophore: *T*_m_ = 68.0 °C [17].

**Figure 1:**
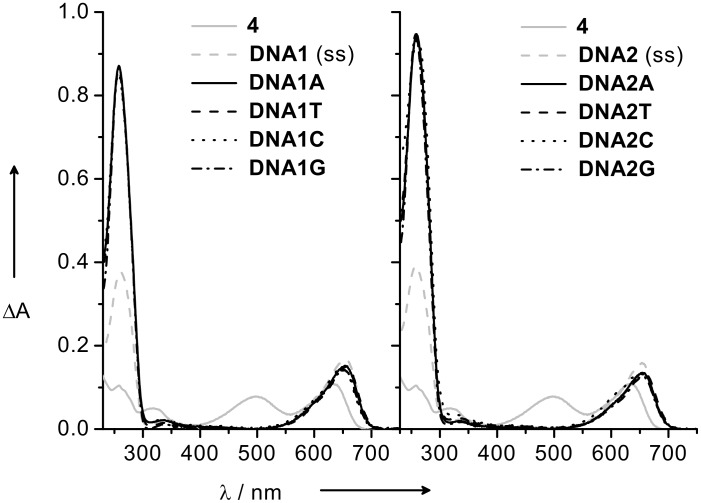
UV–vis absorption spectra of single-stranded **DNA1** and **DNA2**, and the corresponding duplexes **DNA1Y** and **DNA2Y** (2.5 μM) in sodium phosphate buffer (10 mM) of pH 7.0, NaCl (250 mM), λ_exc_ = 610 nm.

The UV–vis absorption properties of all Nile Blue-modified duplexes are remarkably different from the Nile Blue dye in ethanol but remarkably similar within the duplex sets **DNA1Y** and **DNA2Y**. The Nile Blue base surrogate of the modified single strands is displayed by the characteristic absorption in the range between 600 and 550 nm (Figure 1). The observed differences between the Nile Blue dye and the corresponding modified oligonucleotides in the visible range are the loss of one absorption band and a red-shift from 633 nm to 653 nm for the second absorption signal. A further small bathochromic shift of ca. 5 nm is observed upon hybridization of the modified single strands **DNA1** and **DNA2** to the corresponding DNA duplexes. These observations can be attributed to strong excitonic interactions of the chromophore with adjacent DNA bases in the single strands or even more pronounced with adjacent base pairs in the double strands. The base that is placed opposite to the Nile Blue chromophore has no significant influence on the optical properties since the absorption spectra are remarkably similar. The latter result is similar to the *S*-configured linker [16]. All results together indicate once more that it does not matter if an *S*- or *R*-configured acyclic linker system is used to attach this chromophore to DNA.

**Figure 2:**
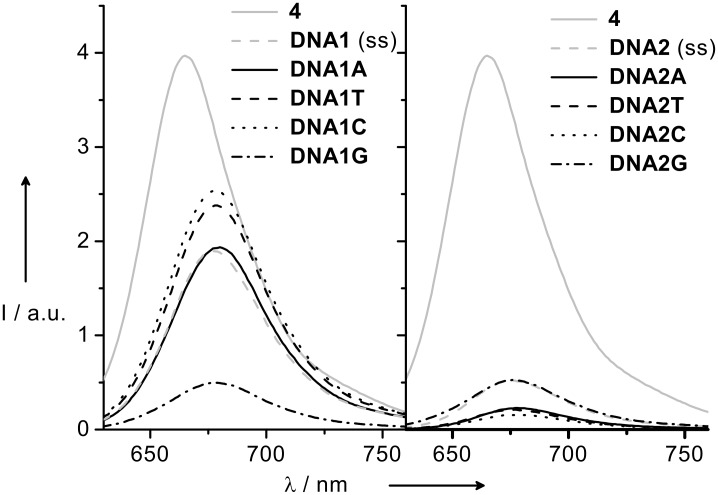
Fluorescence spectra of single-stranded **DNA1** and **DNA2**, and corresponding duplexes **DNA1Y** and **DNA2Y** (2.5 μM) in sodium phosphate buffer (10mM) of pH 7.0, NaCl (250 mM), λ_exc_ = 610 nm.

We interpret the similarity of the absorption properties together with the results from the thermal dehybridization studies, as discussed above, as a result of the intercalation of the Nile Blue dye in duplex DNA. To further explore the optical properties we recorded steady state fluorescence spectra of the Nile Blue-modified single and double strands using an excitation wavelength of 610 nm (Figure 2). The emission maximum of the chromophore is shifted from 665 nm (in case of the isolated dye in ethanol) to 677–679 nm for the modified DNA single and double strands. In contrast to the absorption properties that were pretty similar for all Nile Blue-modified duplexes, fluorescence spectra show the influence of the sequential neighborhood. The quantum yields of the Nile Blue-modified single strands are reduced from 33% for the isolated dye in ethanol to 15% in the single strand **DNA1** and 4% in **DNA2**. Duplexes of the sets **DNA1Y** and **DNA2Y** range from 17 to 22% provided guanines are not placed in the vicinity of the phenoxazinium dye.

The fluorescence is quenched significantly if guanine is present as the counterbase (**DNA1G**), or as part of the adjacent base pairs (**DNA2A**, **DNA2T**, **DNA2C**), or both (**DNA2G**). It becomes clear from a rough estimation of the excited state potential for the Nile Blue dye that guanine oxidation by an photoinduced electron transfer causes the fluorescence quenching. If the singlet–singlet transition energy E_00_ = 1.9 eV is added to the reduction potential E_red_ −0.3 V (vs. NHE), the excited state potential is estimated to be E^*^_red_ = 2.2 eV [17]. Hence, a photooxidation of guanine is highly favorable because the oxidation potential of guanine is only ~1.3–1.4 V [44]. Hence sequence-dependent fluorescence quenching of the Nile Blue dye in DNA was expected and was used as a sensitive tool to compare the electronic interactions of the chromophore with the DNA base stack in order to evaluate the role of chirality of the glycol linker (*R*- vs. *S*-configuration). It is important to point out that a similar emission profile is observed regardless of whether the chromophore has been attached via the *R*-configured linker (this study) or the *S*-configured one [17]. The comparison of quantum yields (Table 1) reflects only very minor differences that are within the experimental error.

**Figure 3:**
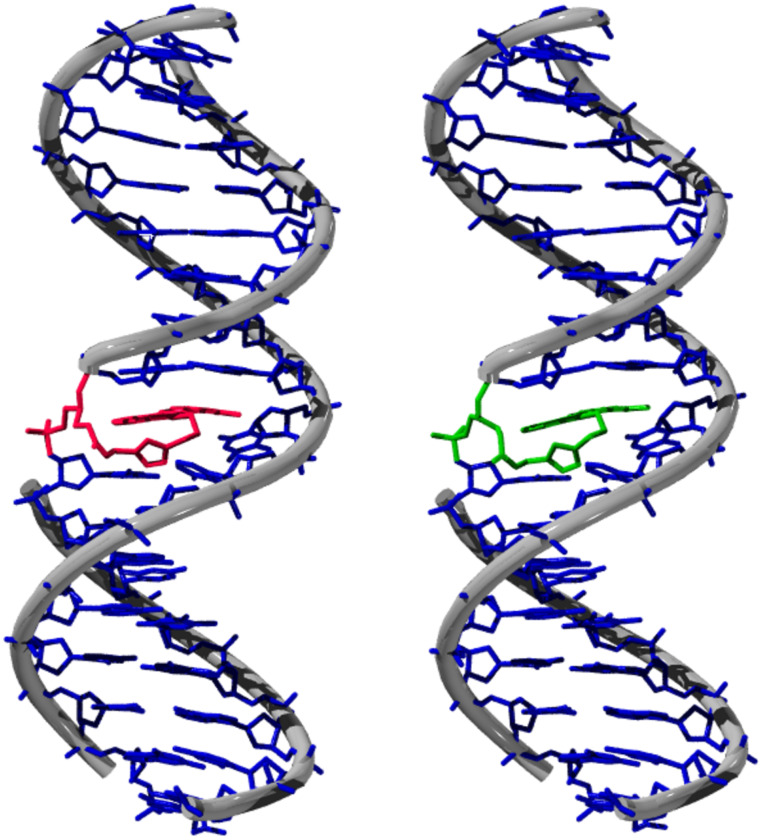
Models for **DNA1A** bearing the (*R*)-3-amino-1,2-propanediol linker (left) and the corresponding duplex with identical sequence but with the (*S*)-3-amino-1,2-propanediol linker (right); AMBER force field, HyperChem 7.5.

It was quite surprising to observe that in contrast to experiments with D- and L-threoninol [39–42] the chirality of the 3-amino-1,2-glycol linker as a substitute for the 2′-deoxyriboside in our studies had no influence on the optical properties of the attached fluorophore. In order to image this result we worked out molecular models for the duplex **DNA1A** (*R*-configuration) and the corresponding duplex bearing the linker in the inverted configuration (*S*). We assumed a base inserted position of the Nile Blue chromophore in both cases (Figure 3). Hence the tether between the 3-amino-1,2-propanediol moiety and the Nile Blue chromophore which consists of two short alkyl chains and the triazolyl group represents the most critical issue for intercalation. From both molecular models it became obvious that this tether is long and flexible enough allowing the Nile Blue chromophore to intercalate in a nearly perfectly stacked position between the adjacent base pairs. This modeling helps to rationalize the experimental result which is the similarity of the optical properties between the two duplexes.

## Conclusion

“Click” chemistry allows the postsynthetic modification of oligonucleotides that were presynthesized using the DNA building block **3** bearing an alkyne group and the azide **4** of the base-labile phenoxazinium dye Nile Blue. The chromophore was incorporated as a DNA base surrogate using (*R*)-3-amino-1,2-propanediol as the linker between the phosphodiester bridges. Two DNA duplex sets were prepared with the phenoxazinium azide **4** (one set carried the chromophore in an A-T environment, the second set in a G-C environment) and characterized by optical spectroscopy. A sequence-dependent fluorescence quenching of the chromophore was applied as a sensitive tool to compare stacking interactions with respect to the chirality of the acyclic linker attachment. In fact, only minor and negligible differences were observed. Melting temperatures, UV–vis absorption properties together with fluorescence quenching properties indicate that the chromophore Nile Blue stacks perfectly between the adjacent base pairs regardless of whether it has been attached via an *S*- or *R*-configured linker. This experimental result can be imaged by geometrically optimized DNA models. Our result is remarkable regarding opposite results with the L-/D-threoninol linkers [37–40] that revealed strong differences in stacking and also in function of attached chromophores depending on the chirality of the linker. We were able to show, however, that it does not matter whether Nile Blue has been attached to a DNA duplex via an *R*- or *S*-configured acyclic glycol linker.

## Experimental

**Materials and Methods.** Chemicals were purchased from Aldrich, Alfa Aesar and Merck. Unmodified oligonucleotides were purchased from Metabion. T.l.c. was performed on Fluka silica gel 60 F_254_ coated aluminium foil. Flash chromatography was carried out with silica gel 60 from Aldrich (60–43 µm). Spectroscopic measurements were recorded in sodium phosphate buffer solution (10 mM) with NaCl (250 mM) at pH 7.0 using quartz glass cuvettes (10 mm). ESI mass spectra were acquired in the central analytical facility of the faculty on a ThermoQuest Finnigan TSQ 7000 in negative and positive ionisation mode. NMR spectra were recorded on a Bruker Avance 300 spectrometer in deuterated solvents that had been dried over basic alumina. Chemical shifts are given in ppm relative to TMS. Absorption spectra and the melting temperatures (2.5 µM DNA, 250 mM NaCl, 10–90 °C, 0.7 °C/min, step width 1.0 °C) were recorded on a Varian Cary 100 spectrometer equipped with a 6 × 6 cell changer unit. Fluorescence spectra were acquired on a Jobin-Yvon Fluoromax 3 fluorimeter in spectral steps of 1 nm and an integration time of 0.2 s. All spectra were recorded with an excitation and emission bandpass of 2 nm and are corrected for Raman emission from the buffer solution.

**Synthesis of 2.** 2-Propyn-1-ol (40 µL, 700  µmol) was dissolved in DMF (5 mL). 1,1′-Carbonyldiimidazole (114 mg, 700 µmol) was added and the solution was stirred at r.t. for 3 h. 3-(4,4′-Dimethoxytrityl)-2-hydroxypropylamine (**1**) (268 mg, 700 µmol) was added and the solution was stirred for another 24 h at r.t. and then evaporated to dryness. The crude product was purified by flash chromatography (CH_2_Cl_2_, 0.1% NEt_3_) yielding a yellow solid (44%). T.l.c. (CH_2_Cl_2_ : MeOH = 100 : 2) *R*_f_ = 0.3. ^1^H NMR (300 MHz, [*d*_6_]-acetone): δ = 7.51–7.48 ppm (m, 2H, arom. DMT), 7.37–7.28 (m, 7H, arom. DMT), 6.89–6.86 (m, 4H, arom. DMT), 6.26 (m, 1H, NH), 4.64 (d, *J* = 2.47, 2H, CH_2_CCH), 4.17 (d, *J* = 5.21, 1H, OH), 3.92–3.86 (m, 1H, CH_2_CHCH_2_), 3.79 (s, 6H, OMe), 3.45–3.37 (m, 1H, CH_2_CHCH_2_), 3.22–3.15 (m, 1H, CH_2_CHCH_2_), 3.13–3.05 (m, 2H, CH_2_CHCH_2_), 2.98 (t, *J* = 2.47, 1H, CCH). MS (FAB): *m/z* (%) 303.1 (100) [DMT]^+^, 475,5 [MH^+^]. HRMS (FAB): M^+^ calc. for C_28_H_30_NO_6_ [MH^+^]: 476.2073; found: 476.2085.

**Synthesis of 3.** Compound **2** (350 mg, 0.74 mmol) was dissolved under argon in dry CH_2_Cl_2_ (5 mL). Dry ethyldiisopropylamine (380 µL, 2.21 mmol) and 2-cyanoethyl-*N,N*-diisopropylchlorophosphoramidite (181 µL, 0.81 mmol) were added. The solution was stirred for 3 h at r.t. Methanol (20 µL) was added to the mixture and stirred for 0.5 h to stop the reaction. The solution was evaporated to dryness and dissolved in dry MeCN (6.1 mL) and applied directly for automated DNA synthesis. T.l.c. (CH_2_Cl_2_ : MeOH = 100 : 2): *R_f_* = 0.6. ^1^H NMR (300 MHz, [*d*_6_]-C_3_D_6_O): δ = 1.06–1.19 (m, 12H, 4_*_Me (^i^Prop)), 2.58 (t, *J* = 6.04, 1H, ≡H), 2.76 (m, 2H), 2.95 (m, 1H), 3.07 (m, 1H), 3.22 (m, 1H), 3.36 (m, 1H), 3.54 (m, 1H), 3.61 (m, 2H), 3.85 (m, 1H), 3.75 (s, 6H, 2_*_OMe), 4.10 (m, 1H), 4.60 (dd, Jz = 2.47, CH_2_≡), 6.20 (m, 1H, NH), 6.83–6.88 (m, 4H, arom.), 7.25–7.35 (m, 7H, arom.), 7.46–7.49 (m, 2H, arom.). ^31^P NMR (121 MHz, [*d*_6_]-acetone): = 150.6.

**Preparation of modified oligonucleotides.** Oligonucleotides were prepared on an Expedite 8909 synthesizer from Applied Biosystems (ABI) using standard phosphoramidite chemistry. Reagents and controlled pore glass (CPG) (1 µmol) were purchased from Proligo. The synthesis of DNA oligonucleotides modified with the acyclic linked acetylene was performed using a modified protocol. Activator solution (0.45 M tetrazole in acetonitrile) was pumped together with the building block (0.15 M in acetonitrile) through the CPG vial. The coupling time was extended to 61 min with an intervening step after 30.8 min for washing and refreshing the activator/phosphoramidite solution in the CPG vial. The CPG vial was flushed with dry acetonitrile after coupling. After preparation, the trityl-off oligonucleotides were cleaved from the resin and deprotected by treatment with concd. NH_4_OH at r.t. for 24 h.

**“Click” ligation.** The azide **4** [18] (114 µL, 10 mM), Cu(I) (17 µL, 100 mM), TBTA (34 µL, 100 mM), each in DMSO : ^t^BuOH = 3 : 1, and sodium ascorbate (25 µL, 400 mM) in H_2_O were added to the oligonucleotide (1 µmol). The reaction mixture was vortexed, shaken overnight at r.t. and then evaporated to dryness using a speedvac. Sodium acetate (100 µL, 0.15 mmol) was added and the mixture stored for 1 h at r.t. Ethanol (1 mL) was added and the mixture vortexed and frozen (−20 °C) overnight. The suspension was centrifuged (13 000 rpm, 15 min) and the supernatant removed. The pellet was washed twice with ethanol (500 µL, 70%) and then dissolved in water (500 µL). Prior to purification by HPLC the DNA was desalted by NAP-5 column (GE Healthcare).

**DNA purification.** The modified oligonucleotides were purified by HPLC on a semipreparative RP-C18 column (300 Å, Supelco) using the following conditions: A = NH_4_OAc buffer (50 mM), pH = 6.5; B = acetonitrile; gradient 0–30% B over 50 min, flow rate 2.5 mL/min, UV–vis detection at 260 and 641 nm. The oligonucleotides were lyophilized and quantified by their absorbance in water at 260 nm on a Varian Cary 100 spectrometer. Duplexes were formed by heating to 90 °C (15 min) followed by slow cooling. MS (ESI): **DNA1**: calc. 5455, found *m/z* = 1362.9 [M/4]^4−^, 1817.7 [M/3]^3−^; ε (260 nm) = 159 090 [mol L^−1^ cm^−1^]; **DNA2**: calc. 5503, found *m/z* =1375.1 [M/4]^4−^, 1833.8 [M/3]^3−^; ε (260 nm) = 164 290 [mol L^−1^ cm^−1^].
